# Recycling Chocolate Aluminum Wrapping Foil as to Create Electrochemical Metal Strip Electrodes

**DOI:** 10.3390/molecules26010021

**Published:** 2020-12-23

**Authors:** Hairul Hisham Hamzah, Nur Hidayah Saleh, Bhavik Anil Patel, Mohd Muzamir Mahat, Saiful Arifin Shafiee, Turgut Sönmez

**Affiliations:** 1 School of Chemical Sciences, Universiti Sains Malaysia (USM), Gelugor 11800, Penang, Malaysia; hidayahsaleh96@gmail.com; 2 School of Pharmacy and Biomolecular Sciences, University of Brighton, Brighton BN2 4GJ, UK; B.A.Patel@brighton.ac.uk; 3 School of Physics and Materials Studies, Faculty of Applied Sciences, Universiti Teknologi MARA, Shah Alam 40450, Selangor, Malaysia; mmuzamir@uitm.edu.my; 4 Kulliyyah of Science, International Islamic University Malaysia, Jalan Sultan Ahmad Shah, Bandar, Indera Mahkota, Kuantan 25200, Pahang, Malaysia; sabs@iium.edu.my; 5 Department of Energy Systems Engineering, Technology Faculty, Karabük University, Karabük 78050, Turkey; turgutsonmez@karabuk.edu.tr; 6 Institut für Technische und Makromolekulare Chemie, RWTH Aachen University, Worringerweg 2, 52074 Aachen, Germany

**Keywords:** conductive chocolate wrapper, aluminum, low-cost electrode, electrochemical metal strip electrode, and sustainability

## Abstract

The development of low-cost electrode devices from conductive materials has recently attracted considerable attention as a sustainable means to replace the existing commercially available electrodes. In this study, two different electrode surfaces (surfaces 1 and 2, denoted as S1 and S2) were fabricated from chocolate wrapping aluminum foils. Energy dispersive X-Ray (EDX) and field emission scanning electron microscopy (FESEM) were used to investigate the elemental composition and surface morphology of the prepared electrodes. Meanwhile, cyclic voltammetry (CV), chronoamperometry, electrochemical impedance spectroscopy (EIS), and differential pulse voltammetry (DPV) were used to assess the electrical conductivities and the electrochemical activities of the prepared electrodes. It was found that the fabricated electrode strips, particularly the S1 electrode, showed good electrochemical responses and conductivity properties in phosphate buffer (PB) solutions. Interestingly, both of the electrodes can respond to the ruthenium hexamine (Ruhex) redox species. The fundamental results presented from this study indicate that this electrode material can be an inexpensive alternative for the electrode substrate. Overall, our findings indicate that electrodes made from chocolate wrapping materials have promise as electrochemical sensors and can be utilized in various applications.

## 1. Introduction

Various widely used commercial electrodes use materials, such as gold (Au), platinum (Pt), and glassy carbon (GC), which are relatively expensive (~US$200 for each electrode), thus making them less accessible for large scale electrochemical studies, particularly in developing countries. Therefore, it is important to develop a low-cost disposable electrode to conduct electroanalytical measurements anywhere in the world. The most common format of an electrode which has been established as a disposal electrode is the screen-printed electrode (SPE). However, the processes to prepare SPE require well-trained staff with an in-depth knowledge of the conditions required for a successful electrode printing [1].

A great deal of effort has been devoted to find a low-cost alternative to conventional electrode materials that force researchers to look at the electrochemical properties of metal scraps [2,3], papers, agricultural by-products, or used batteries [4]. This approach can provide environmental benefits by properly managing recyclable waste materials for significant usage in a variety of advanced materials such as composite materials [5,6], biomaterials [7], and electrochemical materials [8,9]. Thus, this approach supports important derives to reduce waste and explore creative approaches to recycle materials for new and diverse applications. This is a key aspect of the United Nations Sustainable Goals, in which responsible consumption and production is a key aspect [10].

A variety of different waste materials have been used to fabricate electrodes such as disposed screen printed electrodes [11], quantitative filter paper and polyethene terephthalate (PET) from beverage bottles [8,9] and adhesive medical tape [12]. One interesting study carried out by Janegitz and co-workers [8] demonstrated that disposable electrodes can be easily prepared by modifying filter papers or PET with conductive inks to make electrode. All these approaches are cost-effective and provide simple approaches to electrode manufacture. Although all these approaches effectively recycle used materials, they only act as substrates in which additional processing is required to make electrodes. However, very few approaches have taken existing disposed materials that are conductive and utilized them for electrode manufacture and use.

In this study, we explored the potential of using chocolate wrappings to make electrochemical metal strip electrodes. Many chocolate wrappers are made from aluminium metal sheets, which have low resistivity (*ρ* = 3 × 10^−8^ Ωcm) [13], are highly conductive, less toxic, and inexpensive [14], thus making them suitable for making electrodes. Electrodes made from chocolate wrappers were characterized using various electrochemical techniques. We demonstrate that aluminum chocolate wrapper working electrodes have electrochemical activities when monitoring an oxygen reduction reaction (ORR), hydrogen evolution reaction (HER), and oxygen evolution reaction (OER). To show that the fabricated electrode strips could provide an electroactive surface for redox reactions, ruthenium hexamine (Ruhex) redox species was used as a model system. Ruhex was chosen for this work because it is classified as an outer-sphere redox system where the molecules are not forming a chemical bridge with the electrode surface in the course of the redox process.

## 2. Results and Discussions

### 2.1. EDX Measurements for S1 and S2 Electrode Surfaces

**Figure 1:**
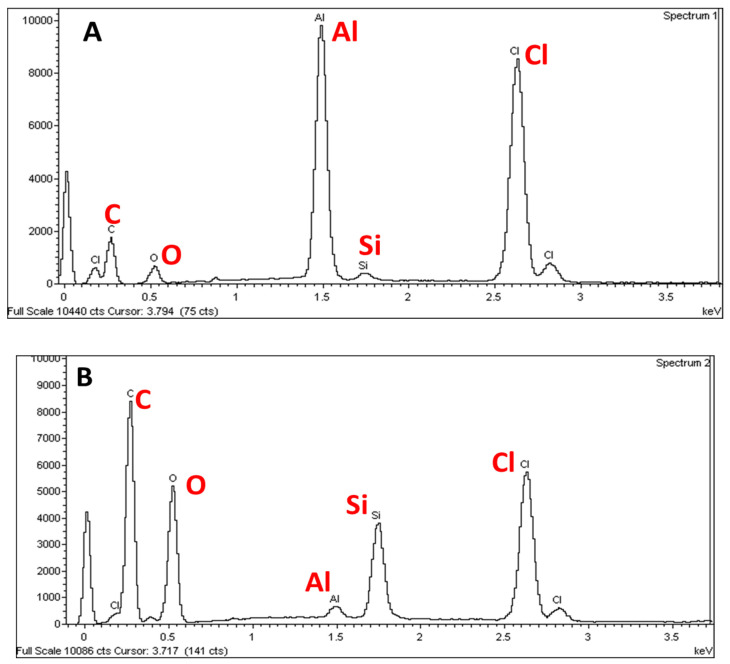
EDX spectra of the S1 (**A**) and S2 (**B**) electrode surfaces. The S1 electrode was fabricated from an inner surface of the wrapper (silver color) whereas the S2 electrode was prepared from an outer surface of the wrapper (gold color).

**Figure 2:**
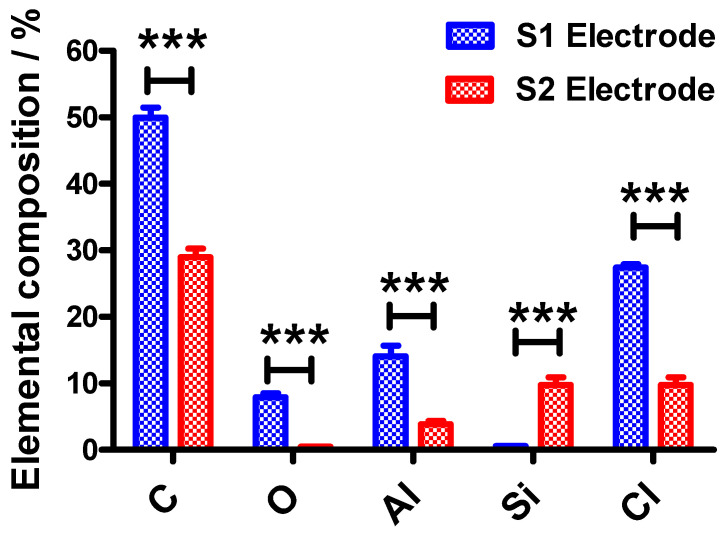
The elemental compositions of the S1 and S2 electrodes showing the percentage of carbon (C), oxygen (O), aluminum (Al), silicon (Si) and chlorine (Cl) which were determined from EDX measurements. Statistical analyses were performed using two-way ANOVA, followed by a Bonferroni test. Data shown as mean ± S.D., *n* = 3 and *** denotes *p* < 0.001.

In order to investigate the superficial amount of the elemental compositions of the S1 and S2 electrodes, EDX measurements were conducted on three different chocolate wrapping aluminum foils that were purchased from different local supermarkets. The EDX spectra are shown in Figure 1 and the elemental compositions that existed at the surfaces are presented in Figure 2.

An intense peak is observed at 1.5 keV on the EDX spectra for the S1 electrode whereas a much less intense peak is observed for S2 electrode which is ascribed to Al. This indicates that the S1 electrode surface contains more Al than the S2 electrode surface and this is in agreement with results obtained using EDX as shown in Figure 2. This could be also attributed to the wrapper colors. The gold color may be attributed to the colorant added for cosmetics and exclusivity purposes. Since the colorant is introduced at the outer surface of the wrapper, the content of Al is lower for the outer surface than for the inner surface of the wrapper. In contrast, the silvery color of the inner surface of the wrapper is due to the higher amount of Al at the surface. From the EDX spectra recorded for both electrodes, using two-way ANOVA followed by Bonferroni post‑hoc test, there were significant differences in the elemental compositions of the S1 electrodes (*p* < 0.001) when compared to the S2 electrodes.

Moreover, the EDX spectra also exhibited some peaks attributed to C and Cl which could be because the wrappers may be coated with polyvinyl chloride (PVC). Some aluminum chocolate wrappers are laminated on the outside or inner side of the foil with PVC to add an extra dimension to a variety of wraps in an assortment and provide a rigid coating but will still retain the twist [15]. In contrast, bi-axially oriented polyethylene terepthalate (BOPET), polypropylene (PP) and polylactic acid (PLA) are also commonly used to coat the aluminum foils [15]. Meanwhile, Si spectra are also observed at 1.75 keV (Figure 1A,B). The presence of the Si peak is due to the addition of Si element to the Al in order to improve the mechanical properties of aluminum chocolate wrapping foil, in particular, to reduce the melting temperature and improve the strength of the wrapper [16]. Thus, from the EDX spectra, it is suggested that lower amount of Si is added to the S1 surface (inner, silver color) compared to the S2 surface (outer, gold color) because the outer layer is more exposed to heat.

### 2.2. CV for S1 and S2 Electrodes in PB Solution (pH 7)

**Figure 3:**
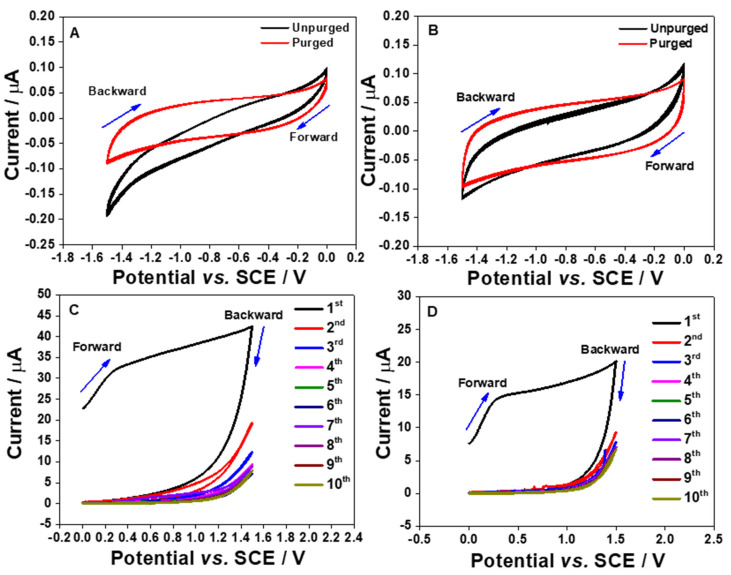
Cyclic voltammograms (10 cycles) of the S1 (**A**,**C**) and S2 (**B**,**D**) electrodes in an aerated and purged PB solutions (pH 7) at 100 mV s^−^^1^. The potential was cycled in the negative region (**A**,**B**) from 0 to −1.5 V vs. SCE and in the positive region (**C**,**D**) between 0 and 1.5 V vs. SCE.

It is quite possible to reduce dissolved oxygen existed in the test solutions by sweeping the potential from positive to more negative potentials if the electrode materials exhibit electrocatalytic activity towards oxygen. As reported by Bučko et al., [17], the standard concentration of aerated O_2_ in aqueous solution is 2.5 × 10^−7^ mol cm^−3^ or 0.25 mM. Thus, it is a common approach for electrochemists to initially deoxygenate the solution before conducting the voltammetric measurements. In this part of the experiment, the CV measurements were monitored in the PB solution without O_2_ saturation as the intention was to investigate the intrinsic behavior of the aerated O_2_ solution towards the fabricated electrodes. Figure 3A,B show the CV currents in the presence and the absence of dissolved O_2_ are very stable even for the 10th cycle. To verify this, by keeping the same approach, the CV response of bare polished gold electrode for aerated O_2_ solution was also monitored as shown in Supplementary Figure S2.

In Figure 3A, the S1 electrode showed an electrocatalytic activity towards the ORR, which was not observed at the S2 electrode (Figure 3B). The ORR onset potential for the S1 electrode is started at approximately −0.6 V vs. SCE (Figure 3A). This could be due to the higher Al content in the S1 electrode when compared to the S2 electrode. This shows the potential of the S1 electrode for probing the dissolved oxygen content. Additionally, from the CVs shown in Figure 3B for the S2 electrode, it is clear that the changes on the CV characteristics as displayed in Figure 3A are connected with O_2_ in the solution, but not with surface reactions.

The electrochemical characteristics of the electrode strips in the positive potential region were also assessed. The potentials were swept from 0 to 1.5 V vs. SCE. A broad oxidation peak can be observed in Figure 3C,D. However, the voltammetric currents are significantly reduced after the first cycle until the oxidation peak completed disappeared. This shape of CV shown in Figure 3C,D has been reported in the literature for the oxidation of Al [18]. The large oxidation peak that is seen on the first scan is contributed from the formation of the Al_2_O_3_ layer at the surface. This is because a layer of Al_2_O_3_ can be easily formed when the aluminum surface makes contact with water or air [18,19]. The surface oxidation process can also take place when the potential is scanned to more positive values. Since the Al content in the S1 electrode is higher than that in the S2 electrode, the oxidation current of the first scan recorded using the S1 electrode is larger than that acquired by the S2 electrode. After the first scan, the oxidation peak is started to decrease significantly, which is most likely due to all the Al atoms that are completely oxidized. The formation of the Al_2_O_3_ layers at the electrode surfaces are suggested to address the occurrence [18]. As a result, the oxidation peaks are completely disappeared on the second cycle of CVs. The formation of Al_2_O_3_ film would give an advantage to the electrode reaction surface as the oxide film could strongly adsorb cations and anions by strong electrostatic fields across the film [20]. Moreover, the Al_2_O_3_ film can be also used to synthesize nanostructured materials, such as nanomesh, nanotubes, and nanowires [13]. 

**Figure 4:**
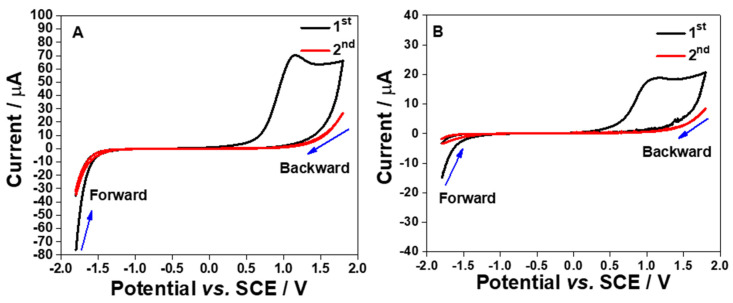
Cyclic voltammograms (1st and 2nd cycles) of the S1 (**A**) and S2 (**B**) electrodes in a purged PB solution (pH 7, 0.1 M) at 100 mV s^−1^. The potential was cycled from −1.8 to 1.8 V vs. SCE.

A potential window study was also conducted in order to determine the working potential range for both S1 and S2 electrodes where a wider potential window was investigated starting from −1.8 to 1.8 V vs. SCE. The results are shown in Figure 4A,B. 

From the CVs, the hydrogen evolution reaction (HER) starts at −1.5 V vs. SCE for both electrodes. Meanwhile, oxide formation peaks can be seen for both electrodes which start at approximately −0.3 V vs. SCE. However, the oxide formation peak can only be observed on the first cycle but not on the subsequent cycles; this could be because the oxide layers are completely formed at the electrode surface as already discussed in the previous paragraph. In contrast, on the second cycle, the current keeps on increasing when cycling the potential to beyond 1.5 V vs. SCE for both electrodes. This electrochemical response could be ascribed to the foot of the oxygen evolution reaction (OER). This means that the working potential range is from ca. −1.5 to 1.5 V vs. SCE.

### 2.3. Effect of CV Scan Rates on the Double-Layer Capacitance (C_dl_)

**Figure 5:**
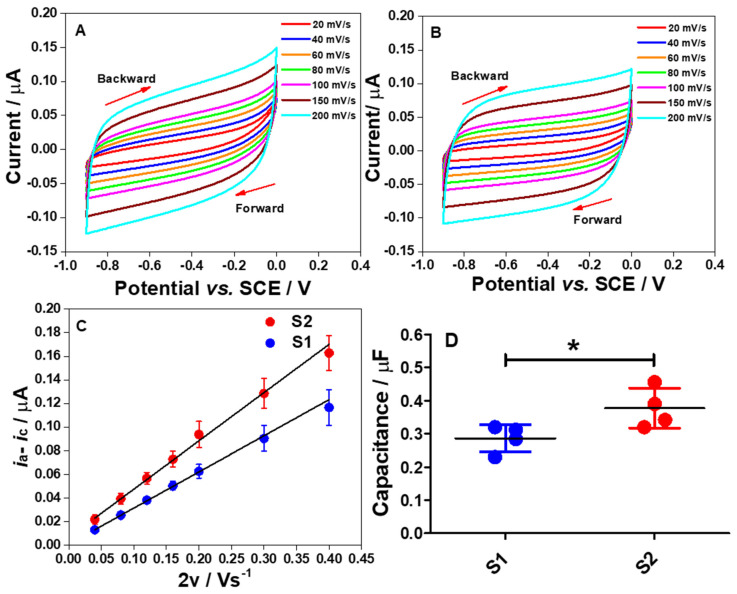
Cyclic voltammograms of S1 (**A**) and S2 (**B**) electrode strips in a purged 0.1 M PB solution of pH 7 at different scan rates from 20 to 200 mV s^−1^. Both electrodes were measured from 0 to −0.9 V vs. SCE. (**C**) The plots of Δ*i* vs. 2*v* at −0.5 V for the S1 and S2 electrodes to determine the *C_dl_*. (**D**) Comparison of the determined *C_dl_* values between the S1 and S2 electrodes. Statistical analysis was performed using an unpaired student *t*-test. Data shown as mean ± S.D., *n* = 4 and * denotes *p* < 0.05.

To evaluate the nature of the double-layer capacitance (*C_dl_*) of the electrode strips in PB solution, the effect of scan rate on the CV measurement in the potential range of 0 to −0.9 V vs. SCE was explored as shown in Figure 5A,B.

The scan rates (*v*) were varied from 20 to 200 mV s^−1^. The results show that the responses are close to the rectangular cyclic voltammograms, demonstrating an excellent electrical double layer capacitance (*C_dl_*) performance and low resistivity, at both high and low scan rates [21]. Thus, it can be concluded that, in PB solution at pH 7, the electrodes provide a highly charged surface profile to attract ions from the bulk solution to the electrode interface in the negative potential region, causing surface charge screening and leading to the formation of an electrical double layer. 

To determine the *C_dl_* values for both electrodes, Equation (6) was used and linear plots of *i_a_* − *i_c_* vs. 2*v* were obtained as shown in Figure 5C. Meanwhile, Figure 5D demonstrates a comparison of the determined *C_dl_* values for S1 and S2 electrodes. Using an unpaired student *t*-test, there was a significant difference in the *C_dl_* values of the S1 electrode when compared to the S2 electrode (*p* < 0.05). Several factors affect the *C_dl_* of the electrode at the interface, such as surface functionality and composition, the geometric surface area of the electrode, surface roughness, and electroactive surface area [22]. However, it is suggested that two main factors can be attributed to this data pattern. First is the surface roughness of fabricated electrodes. Ideally, the capacitive current symmetrically at a specific potential from CV is given as in Equation (1):

(1) $$i_{C}=\frac{i_{a}-i_{c}}{2}$$

where *i_C_* is the capacitive current, *i_a_* is the anodic capacitive current and *i_c_* is the cathodic capacitive current. In our data analyses, *i_C_* was obtained at −0.5 V as mentioned in Section 3.4. As the *C_dl_* values of four different electrodes have already been obtained as shown in Figure 5D, the dependency of the *C_dl_* to the capacitive current flowing at the electrode/electrode interface on the scan rate is theoretically connected as shown in Equation (2) [23]:

(2) $$i_{C}=C_{dl}v$$

where *v* is the scan rate. This dictates that increased the capacitive current (*i_C_*) will increase the *C_dl_*. Interestingly, as the *C_dl_* and *i_C_* can be obtained from Equations (6) and (1), respectively, the real surface area (*A_real_*) of the solid electrodes can be estimated by using a differential equation of the capacitive current as given in Equation (3) [24,25]:

(3) $$i_{C}=A_{real}C_{dl}\frac{\delta_{E}}{\delta_{t}\;}=A_{real}C_{dl}v$$

where the *A_real_* is the real surface area of the solid electrode, *δ_E_* is the potential difference, and *δ_t_* is the time difference. Thus, by using Equation (3), the *A_real_* can be estimated. Therefore, to calculate the *A_real_* from Equation (3), the *i_C_* and *C_dl_* must be initially calculated. As the *A_real_* is essentially used to find the roughness factor (*ρ*) of the electrode surface, the *ρ* can be calculated as expressed in Equation (4) [25]:

(4) $$\rho =\frac{A_{real}}{A_{geo}}$$

where *A_geo_* is the geometric surface area of the electrode. Thus, an increase in the *A_real_* will increase the *ρ* value. The bigger *ρ* value indicates the rougher electrode surface. In order to support this claim, the calculated *i_C_* and obtained *C_dl_* values for the S1 and S2 electrodes were employed in Equation (3) to calculate the *A_real_*. Subsequently, by using Equation (4), the *ρ* values were calculated as shown in Table 1.

**Figure 6:**
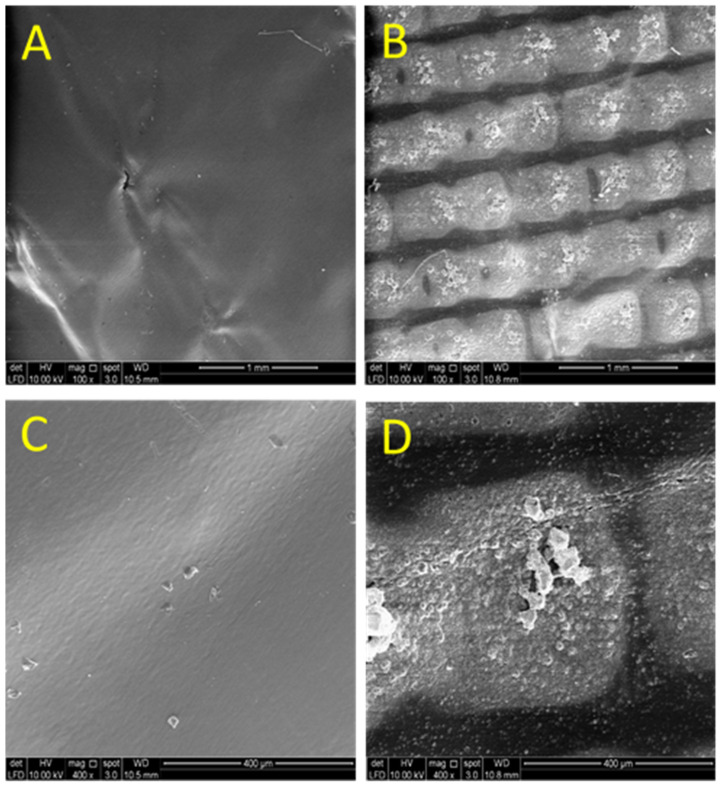
FESEM micrographs of S1 (**A**,**C**) and S2 (**B**,**D**) electrode taken at the surface at different magnifications: (**A**,**B**) 100× & (**C**,**D**) 400×.

This dictates that the surface of the S2 electrode is rougher than the S1 electrode surface. In order to support this statement, the surface morphologies of both electrodes were investigated at different magnifications (100× and 400×) using field emission gun scanning electron microscopy (FESEM; FEI) as displayed in Figure 6A–D. From the obtained images, it shows that the S2 surface is rougher than the S1 surface. The S1 electrode surface is very smooth, whilst the surface of the S2 electrode consists of neatly ordered rows of metal blocks. This morphology surely increases the electrode surface area which may have led to a higher capacitance observed on the S2 electrode. This has been shown in Table 1 and it is following the *C_dl_* for the porous electrode materials as connected by Equation (5) [26]:

(5) $$C=\frac{\varepsilon A}{d}$$

where *A* is the surface area of the electrode, *ε* is the electrolyte dielectric constant and *d* is the distance from the surface of the electrode to the center of the ionic layer. This shows that *C* is proportional to the *A*.

The second main factor affecting the *C_dl_* value of the electrode is the surface composition of the fabricated electrodes. As the percentage compositions of the elements indicated by EDX for both electrodes differ significantly (Figure 2), this could explain why the *C_dl_* of the S2 electrode is higher than the S1 electrode. Thus, it is believed that the aforementioned two factors are mainly ascribed to the different behavior of the *C_dl_* values as shown in Figure 5C,D.

### 2.4. Chronoamperometric Measurements for S1 and S2 Electrodes

**Figure 7:**
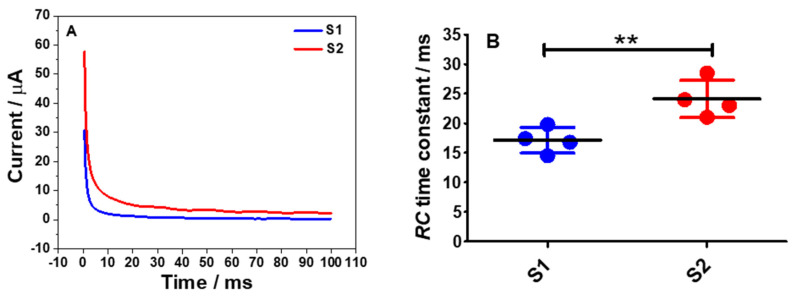
(**A**) Chronoamperograms of S1(blue) and S2(red) electrodes in a degassed 0.1 M PB buffer solution of pH 7 by stepping the potentials from −0.9 to 0 vs. SCE and it was held at the latter potential for 100 ms. (**B**) Comparison of the *RC* time constants calculated from the plots of ln *i* vs. *t*. Statistical analysis was performed using an unpaired student *t*-test. Data shown as mean ± S.D., *n* = 4 and ** denotes *p* < 0.01.

One of the most important parameters which can be determined from the newly fabricated electrode is the *RC* time constant. The *RC* time constant is the time taken for the charging current to flow through the solution with respect to the resistance (*R*) and capacitance (*C*). The most common technique that can be employed to determine the *RC* time constant is chronoamperometry. Figure 7A shows chronoamperograms recorded using S1 and S2 electrodes in a 0.1 M PB solution by stepping the potentials from −0.9 to 0 V vs. SCE and the potential was held at the latter for 100 ms. 

Essentially, for typical chronoamperograms, the charging current or capacitive current decays exponentially and then stabilizes as time progresses at a rate governed by *RC* as can be expressed in Equation (7) [27] and a graph of ln *i* vs. *t* can be plotted. From the plot, a linear regression line for the charging current was obtained and from Equation (7), a straight line equation can be expressed as ln *i_C_* = (−1/*RC*) *t* + ln(∆*E*/*R*), where the gradient value is −1/*RC*. Thus, the *RC* time constant for the S1 and S2 electrodes were calculated accordingly and the results are shown in Figure 7B. From both datasets, there was a significant difference in the *RC* time constant of S1 electrodes (*p* < 0.01) when compared to the S2 electrodes. This data pattern can also clearly be seen on the chronoamperograms of the S1 and the S2 electrodes where the charging current for the S1 electrode took a shorter time to reach the plateau region than the S2 electrode.

### 2.5. Electrochemical Impedance Spectroscopy (EIS) for S1 and S2 Electrodes

**Figure 8:**
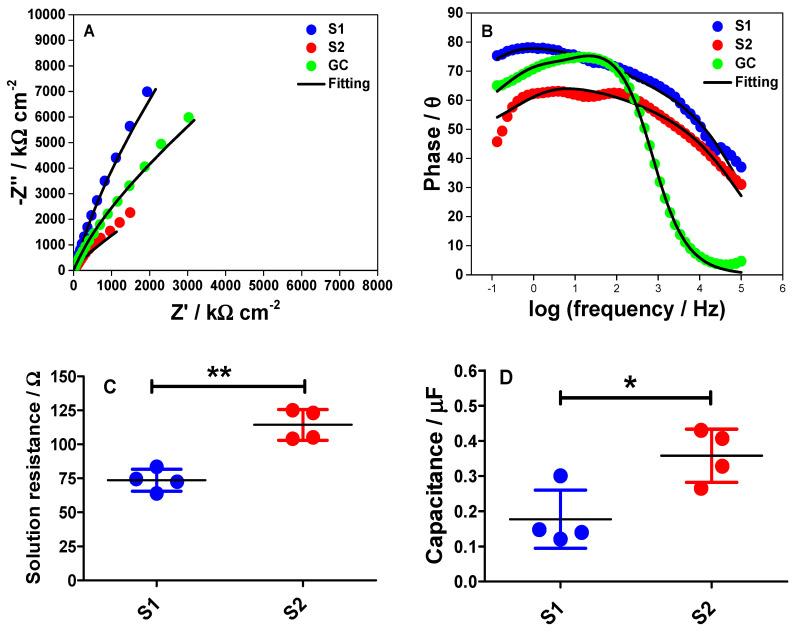
Nyquist (**A**) and Bode (**B**) plots for the GC, S1, and S2 electrodes at −0.6 V vs. SCE in phosphate buffer solution (pH 7). Experimental data are depicted in filled circles whereas the solid lines are fitting to the equivalent circuit model as proposed in Figure S3. The measurements were made at a range of frequency from 100 kHz to 0.1 Hz and the modulation amplitude of 5 mV. (**C**) Comparison of the solution resistance (*R_S_*) for the S1 and S2 electrodes, determined from the EIS fittings. (**D**) Comparison of the obtained *C_dl_* values between the S1 and S2 electrodes from the EIS fittings. Statistical analyses were performed using an unpaired student *t*-test. Data shown as mean ± S.D., *n* = 4, * denotes *p* < 0.05 and ** denotes *p* < 0.01.

To further understand the interface characteristics of the S1 and S2 electrodes, EIS was conducted in a PB solution at −0.6 V vs. SCE where the employed potential is close to the potential value used in estimating the *C_dl_* values from the CVs. Also, a freshly polished bare GC electrode was utilized as a control experiment in order to find the best equivalent circuit for EIS fittings. In this measurement, a simple equivalent circuit (*RC*) for non-faradaic impedance was assumed where the capacitance (C) and the resistance (*R*) are connected in series. Figure 8A shows the Nyquist plots for the GC, S1, and S2 electrodes, by plotting the imaginary part of impedance (−Z′′) as a function of its real component (Z′) over a wide frequency range from 100 kHz to 0.1 Hz. The frequency in Figure 8A increases from the top right to the bottom. From the Nyquist plots, it can be concluded that the resistance and capacitance are non-uniformly distributed at the S1 and S2 electrode surfaces. This has been clearly shown in the FESEM images (Figure 6A,B). Meanwhile, Figure 8B shows the Bode plots of the GC, S1, and S2 electrodes at −0.6 V.

The Bode plot is fairly important as it can give us some information at three different frequency regions at the interface. In the high-frequency region, the Z’ is independent of the frequency with the phase angle (*θ*) values near to or at 0. This behavior is usually due to the resistance of the electrolyte solution between the working and the reference electrodes whereas, in the medium frequency region, a linear relationship can be observed. This region corresponds to the double-layer capacitance behavior at the electrode/electrolyte interface. The third region can be observed at the low-frequency range where the phase reaches the maximum value. Then, the phase starts to decrease and this is caused by the accumulation of ions at the electrode surface [28,29].

According to data in Figure 8B, the magnitude of the phase angle for the S2 electrode decreases. Interestingly, our data trend complements the *RC* time constant calculated from the chronoamperometric measurements (Figure 7B). This trend implies that the double-layer charging and discharging processes are slower on the S2 electrode than at the S1 electrode surface. Moreover, the different characteristics on the Nyquist plots between the S1 and the S2 electrode surfaces can also indicate that the polar properties of the two electrode surfaces may be different during the EIS measurements.

Both Nyquist and Bode plots were then fitted to the equivalent circuit as displayed in Supplementary Figure S3. Although we assumed that our measurements follow an ideal *RC* equivalent circuit, it is important to note that the real electrochemical system never follows such ideal double-layer charging. The Nyquist plots in Figure 8A have already indicated that the electrodes do not precisely follow the ideal series *RC* circuit characteristic. In order to acquire the best fitting for both datasets, as well as the smallest error values for the fittings, Warburg impedance (*W*) and constant phase element (*CPE*) were used in the equivalent circuit as illustrated in Figure S3. The presence of *W* in the circuit may correspond to the diffusion process of protons and electrolyte ions towards the electrode surface within the diffuse layer in the 0.1 M PB solution. Meanwhile, the *CPE* element in the circuit represents the heterogeneity of the electrode surface. The error values for *R_s_* and *C_dl_*, from the fittings, are shown in Table 2.

Based on Table 2, the proposed equivalent circuit for the EIS measurements fits well to the Nyquist plot of the bare GC electrode and gives small errors for the parameter values. When employing the fitting of the equivalent circuit to the EIS data obtained using the S1 and the S2 electrodes, the fitting also fits well to the EIS spectra of the S1 electrode. However, the fitting does not fit well to the EIS spectra of the S2 electrode and this could be because the surface roughness is not uniform throughout the S2 surface. This reflects a bigger error obtained for *C_dl_* value for S2 electrode from the fitting of the EIS spectrum. Interestingly, this indicates that the proposed equivalent circuit is the best circuit that suits the interface behavior of our electrochemical measurements during the experiments. Moreover, based on the *R_s_* values obtained from the EIS fittings, a graph to show each point of the *R_s_* acquired from four different S1 and S2 electrodes was plotted as shown in Figure 8C. The data show that there was a significant difference in the *R_s_* value for the S1 electrode (*p* < 0.01, *n* = 4) when compared to the S2 electrode. Again, this data trend is in accordance with the *RC* time constant calculated from the chronoamperometric measurements (Figure 7B). In contrast, the capacitance values for S1 and S2 electrode obtained from the EIS fitted data were also statically analyzed as shown in Figure 8D. Remarkably, the dataset shown is in the same trend with the capacitance value obtained from CV (Figure 5D), where there was a significant difference in the capacitance of the S1 electrode (*p* < 0.01, *n* = 4) when compared to the S2 electrode.

### 2.6. Electrochemical Responses of S1 and S2 Electrodes toward the Redox Reactions of Ruthenium Hexamine (Ruhex)

**Figure 9:**
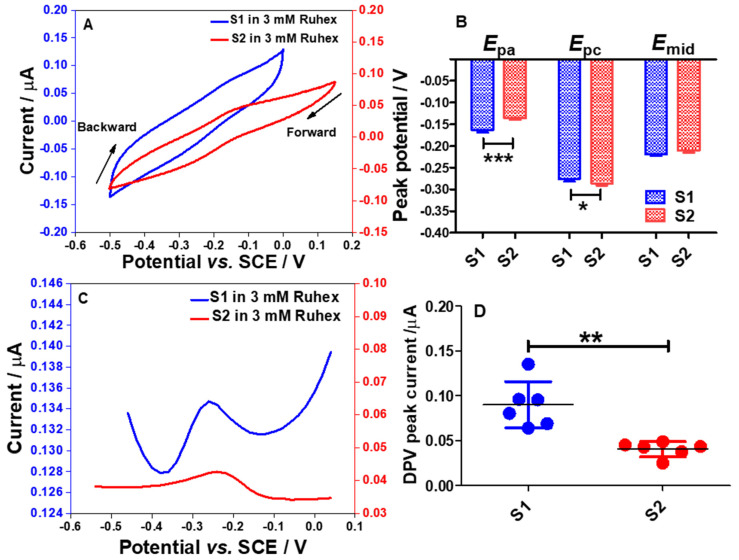
Cyclic voltammogram responses (3rd cycle) recorded using electrode strips; S1 and S2 electrodes (**A**) in 3 mM Ruhex, dissolved in 0.1 M PB solution, by cycling the potentials from 0.2 to −0.5 V vs. SCE at 100 mV s^−1^. (**B**) Comparison of the *E*_pa_, *E*_pc_ and *E*_mid_ between S1 and S2 electrodes. Statistical analyses were achieved using two-way ANOVA, followed by a Bonferroni test. Data shown as mean ± S.D., *n* = 4, * denotes *p* < 0.05 and *** denotes *p* < 0.001. (**C**) Differential pulse voltammograms (DPVs) obtained in the 3 mM Ruhex solution, by scanning the potentials between −0.6 and 0.2 V vs. SCE at modulation amplitude of 50 mV, step potential of 20 mV, modulation time of 50 ms and interval time of 500 ms. (**D**) Comparison of DPV peak currents between S1 and S2 electrodes. Statistical analysis was performed using an unpaired student *t*-test. Data shown as mean ± S.D., *n* = 6 and ** denotes *p* < 0.01.

The redox peaks were seen when performing cyclic voltammetry in 3 mM Ruhex solution using the S1 and the S2 electrodes as shown in Figure 9A. From voltammetric responses, as for the S1 electrode, the *E*_pa_ and *E*_pc_ of [Ru(NH_3_)_6_]^3+^/[Ru(NH_3_)_6_]^2+^ redox molecules were found to be −0.163 ± 0.0059 V and −0.275 ± 0.0067 V, respectively. The mid potential (*E*_mid_) of [Ru(NH_3_)_6_]^3+^/[Ru(NH_3_)_6_]^2+^ redox processes on the S1 electrode was calculated to be −0.219 ± 0.0035 V. Meanwhile, the values of *E*_pa_, *E*_pc_, and *E*_mid_ for the S2 electrode were calculated to be −0.136 ± 0.0044, −0.286 ± 0.0051 V, and −0.210 ± 0.0045 V, respectively. This data pattern can be seen in Figure 9B. The bar graph shows that the S1 electrode demonstrates a significant shift in the *E*_pa_ (*p* < 0.001) and *E*_pc_ (*p* < 0.05) when compared to the S2 electrode. This result is attributed to the different morphologies of the electrode surfaces associated with the electrode composition as already discussed in Section 2.3. However, there was no significant difference in the *E*_mid_ values for both electrodes.

This indicates that the S1 and S2 electrode surfaces can provide electroactive surfaces for redox processes of [Ru(NH_3_)_6_]^3+/2+^. Although both electrode surfaces may undergo the formation of Al_2_O_3_ film, the surface could still be able to provide electroactive sites for the reduction of Ruhex. In order to enhance the electrochemical signal for the reduction of Ruhex, a DPV technique was employed as the capacitive current can be eliminated. The differential pulse voltammograms (DPVs) are shown in Figure 9C. Interestingly, the DPV peaks for the reduction of Ruhex are more pronounced than the peaks shown in the CVs. From the DPV peak currents recorded using the S1 and S2 electrodes, a graph was plotted to show the individual point of the DPV peak currents from six different electrodes as displayed in Figure 9D. The data show that the S1 electrode could provide a better electroactive surface than the S2 electrode for the redox reactions of [Ru(NH_3_)_6_]^3+/2+^. In addition, there is a significant difference in the DPV peak currents for S1 electrodes (*p* < 0.01, *n* = 6) when compared to the S2 electrodes. This data trend was expected due to the higher content of Al in the S1 electrode as compared to the S2 electrode. However, the S2 electrode has a rougher surface than the S1 electrode as shown in the FESEM images which should result in a higher current since the real surface area of the S2 electrode is larger than that of the S1 electrode (Table 1) but this is not the case. This implies that the electrode composition is far superior to the morphology of the electrode surface in dictating the electrochemical performance of the electrodes. Overall, the CV and DPV responses for Ruhex reduction gave an important insight about these particular electrode materials to conduct further study, particularly in investigating the electron transfer kinetics and diffusion behavior of Ru(NH_3_)_6_^3+/2+^ at the S1 and S2 interfaces. It would also be interesting to probe other fast electron transfer outer-sphere redox systems.

## 3. Material and Methods

### 3.1. Chemicals 

Sodium phosphate monobasic (NaH_2_PO_4_), sodium phosphate dibasic (Na_2_HPO_4_), sodium hydroxide (NaOH), and hexaamineruthenium(III) chloride were purchased from Bendosen Laboratory Chemicals (Selangor, Malaysia), Fisher Scientific Malaysia (Shah Alam, Malaysia), Sigma-Aldrich Malaysia (Kuala Lumpur, Malaysia), and Alfa Aesar (Ward Hill, USA), respectively. The chemicals were used without further purification. All solutions employed during the experiments were prepared using deionized (DI) water obtained from a Millipore Direct-Q3 water purification system with a resistivity of 18.2 MΩ cm at 25 °C. Phosphate buffer (PB) of pH 7 ± 0.3 was prepared by mixing a 0.1 M sodium phosphate monobasic solution and a 0.1 M sodium phosphate dibasic solution until the desired pH was obtained. The pH was determined using a pH meter purchased from Eutech Instruments (Ayer Rajah, Singapore).

### 3.2. Fabrication of the Electrode Strips

**Figure 10:**
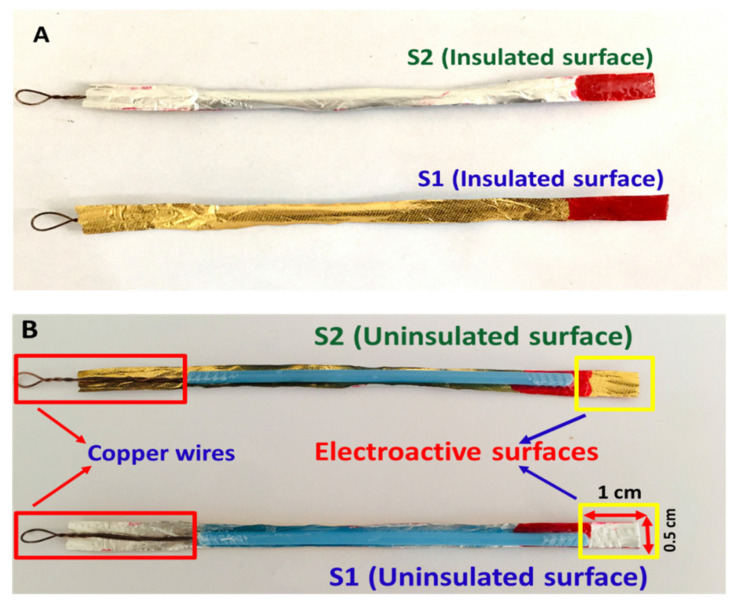
The insulated (**A**) and uninsulated (**B**) surfaces for the S1 and S2 electrodes, fabricated from the chocolate aluminum wrapping foils where the geometric surface areas for both electrodes are 0.5 cm^2^. Both the uninsulated surfaces will be used as electroactive surfaces for electrochemical characterization.

Chocolate wrappers were obtained by purchasing hazelnut chocolates from a local supermarket, manufactured by Ferrero SpA. The wrappers were then wiped with DI water and subsequently with ethanol. They were then rinsed with DI water and left to dry. The chocolate wrappers were cut into small pieces with the dimensions of 8 × 0.5 cm. Then, the strips were affixed to cotton bud sticks using super glue (Elephant) as structural support. The wrappers have two surface sides with each side having a different color, gold and silver. The inner surface of the wrapper is a silver color while the outer surface is a gold color. The silver and the gold sides are denoted as S1 and S2, respectively. To estimate the superficial amount of the elements presented on both sides of the electrode surfaces, energy-dispersive X-ray spectroscopy (EDX; Oxford Instruments, Abingdon, UK) measurements were carried out. To assess the electrochemical properties of one side of the electrode, the other side of the metal strip was painted with nail polish (Bloop), purchased from Guardian Pharmacy to insulate the area from participating in the electrochemical processes. The top part of the side that is taking part in the electrochemical process was also painted with the nail polish leaving only 1 × 0.5 cm dimensions (obtaining 0.5 cm^2^ geometric surface area of the electrode) to control the exposed area of the electrode strips when immersing the electrodes into the electrolytes as shown in Figure 10A,B. The painted side of the electrode was then dried using the heat from the sun. Then, a copper wire was attached to the end of the electrode strip using super glue (Elephant) to make a better connection between the electrode and a crocodile clip. It is important to note that the S1 and S2 surfaces were characterized individually by cyclic voltammetry (CV), chronoamperometry, electrochemical impedance spectroscopy (EIS), and differential pulse voltammetry (DPV).

### 3.3. Electrochemical Characterizations of the Electrode Strips

The prepared electrode strips (S1 and S2) were electrochemically characterized using CV. The CV measurements were performed using an Autolab PGSTAT204 potentiostat/galvanostat (Ecochemie, Utrecht, The Netherland or BASi EC Epsilon^TM^, West Lafayette, IN, USA). A three-electrode system was employed where an electrode strip, a platinum mesh, and a saturated calomel electrode (SCE) were utilized as a working electrode, a counter electrode, and a reference electrode, respectively. The counter electrodes were sterilized with flame prior to usage to remove any contaminants. PB solutions (pH 7, 0.1 M) were used as the supporting electrolyte during the electrochemical measurements. Prior to conducting the CV measurements, a PB solution was purged with nitrogen (N_2_) gas for 30 min, unless otherwise stated, to remove any dissolved oxygen that could affect the electrochemical results. Meanwhile, a blanket of N_2_ was formed on the surface of the solution by maintaining a slow stream of the inert gas over the surface to prevent oxygen from seeping into the solution. Finally, the electrodes were characterized using a widely known redox probe of hexaamineruthenium(III) chloride (Ruhex). A 3-mM Ruhex solution was prepared in 0.1 M PB solutions (pH 7). The use of Ruhex in this work is required since it is an outer sphere redox system, which is a surface-insensitive system. 

### 3.4. Data Analysis

The electrochemical data were treated and analyzed using Origin 9.1 software. The anodic and cathodic peak potentials of Ruhex were obtained from CV measurements by performing a background subtraction from the CVs. The procedure was done by selecting baseline points on either side of the faradaic peaks (anodic or cathodic) and then using a B-spline interpolation routine to estimate the background current in each CV. After the subtraction procedure, the *E*_pa_ and *E*_pc_ were determined as shown in Supplementary Figure S1. Furthermore, the mid potential (*E*_mid_) can be obtained by calculating it as *E*_mid_ = (*E*_pa_ + *E*_pc_)/2. All statistical differences between the datasets for S1 and S2 electrodes were analyzed using an unpaired student *t*-test and Bonferroni test on GraphPad Prism 5.0. The EIS fittings were performed using NOVA 1.11 software (Metrohm Autolab). The data were presented as mean ± standard deviation (SD) and *p* < 0.05 was considered as significant.

To calculate double-layer capacitance (*C_dl_*) of the electrode strips via CV measurements, from the CVs at different scan rates, the *C_dl_* for each electrode can be essentially determined by using Equation (6) [30,31].

(6) $$\;\;\;\;\;\;C_{dl}=\frac{i_{a\;}-\;i_{c}}{2v}$$

Then, by plotting a graph of *i_a_* − *i_c_* at −0.5 V against 2*v*, the *C_dl_* values can be determined from the linear slopes.

On the other hand, to determine the *RC* time constant of the electrodes via chronoamperometric measurements, Equation (7) was used [27].

(7) $$i_{C}=\frac{\Delta E}{R}\;exp^{\frac{-t}{RC}}$$

where *i_C_* is the charging current, ∆*E* is the potential step, *R* is the solution resistance, *C* is the double-layer capacitance, and *t* is the time. To obtain the *RC* time constant values for S1 and S2 electrodes, a graph of ln *i* vs. *t* was plotted.

## 4. Conclusions and Outlook

In this study, electrochemical metal strip electrodes were successfully fabricated from conductive chocolate wrappers with aluminum content. Overall, the S1 electrode exhibited better electrochemical performances compared to the S2 electrode, indicating that the S1 electrode surface offers high conductivity, low resistivity, and more electroactive sites than the S2 electrode. In addition, the electrode composition is far superior to the morphology of the electrode surface in dictating the electrochemical performance of the electrode strip. From the fundamental findings of this study, many possibilities could be explored in our future studies in order to improve the performance of the electrode strips. Notably, the use of chocolate aluminum wrapping foil waste as electrode substrates can provide new strategy in recycling and reusing the waste materials. It could play a significant role in supporting the sustainability agenda, as created by the United Nations (UN). The potential practice that was presented in this study can surely avoid the discarding and burning of chocolate aluminum wrapping foil wastes to the landfills that may result in reducing land, water, and air populations. This approach could be employed as a recycling practice to support reducing the exploitation and degradation of waste in our environment.

## Figures and Tables

**Table 1:** The calculated *A_real_* and *ρ* using Equations (3) and (4) for the four different S1 and S2 electrodes at a scan rate of 0.12 V s^−1^. The geometric surface area of the electrode is 0.5 cm^2^.

Electrode	Parameter	Electrode Number				Mean ± S.D
		1	2	3	4	
S1	*A_real_* (As/Fv)	0.48	0.37	0.50	0.59	0.49 ± 0.090
	*ρ*	0.96	0.74	1.00	1.18	0.97 ± 0.18
S2	*A_real_* (As/Fv)	0.64	0.51	0.68	0.72	0.64 ± 0.091
	*ρ*	1.28	1.02	1.36	1.44	1.28 ± 0.18

**Table 2:** Parameter values and errors for *R_s_* and *C_dl_*, determined by EIS fittings following an equivalent circuit as displayed in Figure S3.

Parameter	GC		S1		S2	
	Value	Error/%	Value	Error/%	Value	Error/%
Solution resistance (*R_s_*)	277.32 Ω	0.58	74.54 Ω	5.31	104.42 Ω	8.21
Double layer capacitance (*C_dl_*)	1.42 μF	2.56	0.30 μF	6.69	0.41 μF	17.17

## Supplementary Materials

The following are available online, Figure S1: The anodic and cathodic currents for the S1 and S2 electrodes in 3 mm Ruhex (as shown in Figure 9A) after performing background subtraction in Origin 9.1 software by using a B-spline interpolation routine to estimate the background current in each CV. Then, the *E*_pa_, *E*_pc_ and *E*_mid_ can be determined, Figure S2: Cyclic voltammograms (10 cycles) of the bare gold electrode in an aerated and purged PB solutions (pH 7) at 50 mV s^−1^. The potential was scanned from 0 to −0.7 V vs. SCE and the geometric electrode surface area is 0.0341 cm^2^, Figure S3: Suggested equivalent circuit model, utilized in convergently fitting the Nyquist and Bode plots from non-Faradaic impedance measurements for fresh polished bare GC, S1 and S2 electrodes. *R_s_* is the solution or electrolyte resistance, *C_dl_* is the electric double layer from electrolyte ions, *W* is the Warburg impedance, *CPE* is the constant phase element and *R_i_* is the internal resistance between the diffuse layer and electrode surface.

## Sample Availability

Samples of the fabricated electrode strips are available from the authors.
