# Supplementary material for: Recycling Chocolate Aluminum Wrapping Foil as to Create Electrochemical Metal Strip Electrodes
Source: Molecules. 2020 Dec 23;26(1):21. doi: 10.3390/molecules26010021 (PMC7793067; doi:10.3390/molecules26010021)
Supplement: Supplementary file 1 [file molecules-26-00021-s001.pdf]

Recycling chocolate aluminum wrapping foil as to create electrochemical metal strip electrodes

Hairul Hisham Hamzah*[1], Nur hidayah Saleh[1], Bhavik Anil Patel[2], Mohd Muzamir Mahat[3], Saiful Arifin Shafiee[4], Turgut Sönmez[5,6]

[1]School of Chemical Sciences, Universiti Sains Malaysia (USM), 11800, Gelugor, Penang, Malaysia.
[2]School of Pharmacy and Biomolecular Sciences, University of Brighton, Brighton, BN2 4GJ, United Kingdom
[3]School of Physics and Materials Studies, Faculty of Applied Sciences, Universiti Teknologi MARA, 40450 Shah Alam, Selangor, Malaysia
[4]Kulliyyah of Science, International Islamic University Malaysia, Jalan Sultan Ahmad Shah, Bandar, Indera Mahkota, 25200 Kuantan, Pahang, Malaysia.
[5]Department of Energy Systems Engineering, Technology Faculty, Karabük University, 78050 Karabük, Turkey
[6]Institut für Technische und Makromolekulare Chemie RWTH Aachen University, Worringerweg 2, 52074 Aachen, Germany

*Corresponding author
E-mail address: hishamhamzah@usm.my

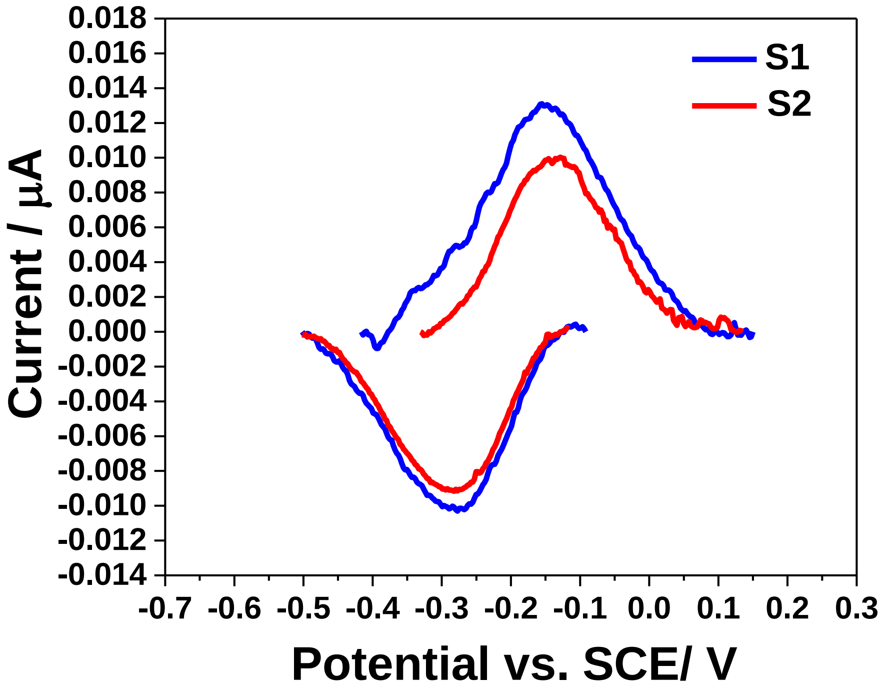

Figure S1: The anodic and cathodic currents for the S1 and S2 electrodes in 3 mm Ruhex (as shown in Figure 9A) after performing background subtraction in Origin 9.1 software by using a B-spline interpolation routine to estimate the background current in each CV. Then, the E_{pa}, E_{pc} and E_{mid} can be determined.

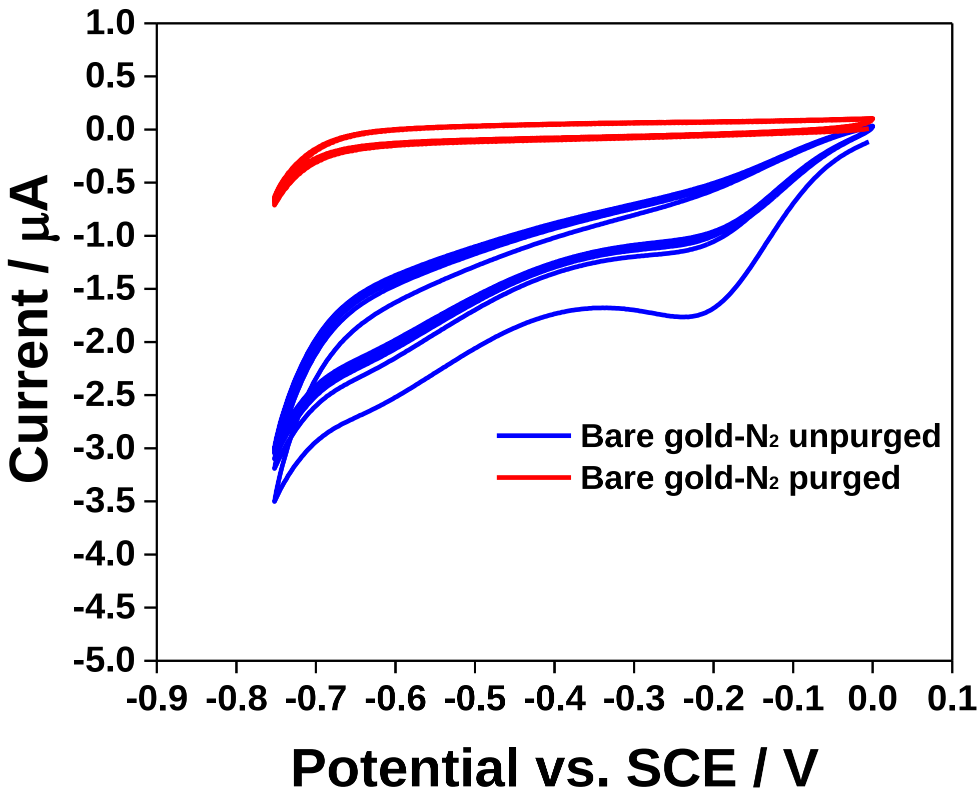

Figure S2: Cyclic voltammograms (10 cycles) of the bare gold electrode in an aerated and purged PB solutions (pH 7) at 50 mV s^{-1}. The potential was scanned from 0 to -0.7 V *vs.* SCE and the geometric electrode surface area is 0.0341 cm^2.

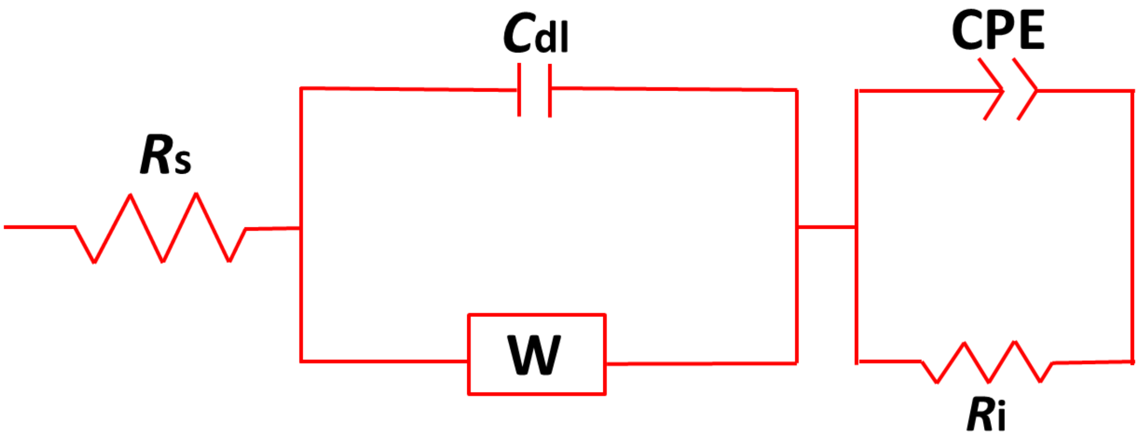

Figure S3: Suggested equivalent circuit model, utilized in convergently fitting the Nyquist and Bode plots from non-Faradaic impedance measurements for fresh polished bare GC, S1 and S2 electrodes. R_s is the solution or electrolyte resistance, C_{dl} is the electric double layer from electrolyte ions, *W* is the Warburg impedance, *CPE* is the constant phase element and R_i is the internal resistance between the diffuse layer and electrode surface.
